# SRGAN-Based Joint Super-Resolution and Denoising for Mitigating Geometric and Topological Biases in Fine-Grained Electron Backscatter Diffraction Images

**DOI:** 10.3390/nano16100583

**Published:** 2026-05-10

**Authors:** Dong Li, Xiaohua Chen, Yongwei Wang

**Affiliations:** 1 State Key Laboratory for Advanced Metals and Materials, University of Science and Technology Beijing, Beijing 100083, China; xtlidong@outlook.com; 2 Collaborative Innovation Center of Steel Technology, University of Science and Technology Beijing, Beijing 100083, China; yw_wang@ustb.edu.cn

**Keywords:** electron backscatter diffraction, super-resolution, denoising, grain statistics, quantitative microstructure analysis

## Abstract

Accurate microstructural characterization for materials with grain sizes ranging from tens to hundreds of nanometers is often constrained by insufficient resolution and noise, leading to distorted statistics. In this study, we constructed a simulated dataset incorporating noise induced by effective resolution based on experimental electron backscatter diffraction (EBSD) characteristics and proposed an SRGAN-based method for simultaneous super-resolution and denoising. Compared with conventional interpolation-based denoising methods widely adopted in EBSD experiments, the proposed framework effectively alleviates the underestimation of grain size and grain boundary number distributions, and more faithfully recovers microstructural geometry and topology. Quantitatively, conventional methods cause an average of approximately 16.03% of the grains to shift toward smaller size bins, whereas the SRGAN-based approach reduces this proportion to about 3.35%. In addition, the SRGAN-based method shows a 2.58% improvement in the accuracy of grain boundary counts compared with conventional method, effectively mitigating statistical distribution distortion. Moreover, although trained exclusively on simulated images, the network performs effectively on experimental images, demonstrating practical applicability for microstructural analysis.

## 1. Introduction

The microstructure of materials plays a critical role in determining their mechanical, physical, and functional properties, making accurate microstructural characterization essential for reliable performance prediction [1,2,3]. Electron backscatter diffraction (EBSD), which predominantly relies on the Hough transform to analyze diffraction Kikuchi bands, is the most common practical method for determining grain size, phase composition, structural anisotropy, and residual strain [4,5,6]. However, EBSD data are constrained by limited resolution, which can alter geometric and topological characteristics.

In addition to the insufficient resolution resulting from the trade-off between accuracy and data acquisition time, the inherent spatial resolution of EBSD also constrains data precision, particularly for fine-grained microstructures, thereby compromising the reliability of statistical analysis. For EBSD data derived from Kikuchi bands analyzed via the Hough transform, the spatial resolution is distinguished as physical resolution and effective resolution. The physical resolution for a given electron beam size is primarily governed by the interaction volume of the electron beam with the sample, which generally decreases as the atomic number of the material increases [7]. The effective resolution is fundamentally determined by the algorithmic capability of the indexing software to discriminate adjacent grains, phases, or twin boundaries [7,8,9]. It was found that the effective resolution was only around one-third of the physical resolution [10]. When the electron beam scans a grain boundary, overlapping Kikuchi patterns arise as orientation information is simultaneously acquired from both adjacent grains [11]. In the Kikuchi bands mixture mode, if the diffraction pattern intensity of an orientation exceeds the recognition threshold (e.g., 60% or 70%), the analysis software can assign that orientation to the pixel; otherwise, the pixel is marked as “unindexed” [9,12]. It is worth noting that these unindexed pixels belong to systematic noise rather than sample preparation defects, and therefore possess physical significance. For samples without residual stress, the indexed rate is directly proportional to the grain size and inversely proportional to the effective resolution of the material [13]. Despite the emergence of Integrated Digital Image Correlation and spherical indexing for resolving overlapping Kikuchi bands [14,15], the requirement for separate purchase of advanced tools like MapSweeper means that the majority of researchers continue to rely on conventional Hough transform-based indexing [16].

**Figure 1:**
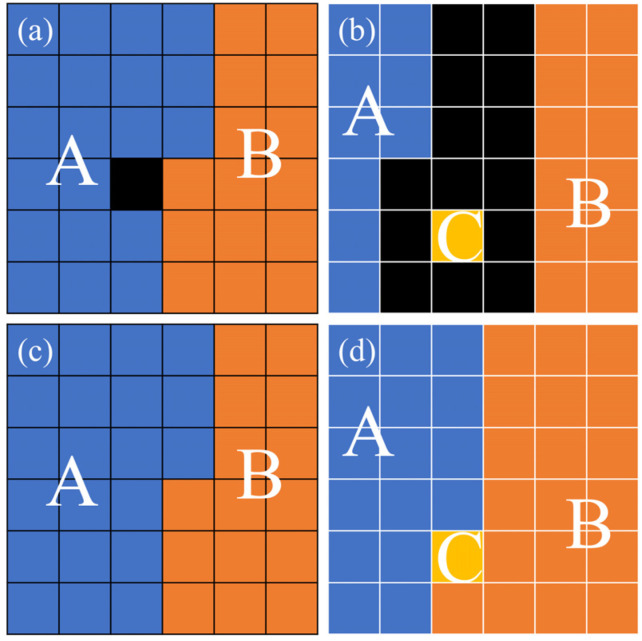
Schematic illustration of denoising in EBSD maps. (**a**) Case without small grains. (**b**) Case including a small grain. (**c**,**d**) Denoised results corresponding to (**a**) and (**b**), respectively. The distinct colors (A, B, and C) denote individual grains, whereas the black areas indicate noise pixels.

In data analysis, applying noise reduction is typically the first step. One of the most common denoising methods works by assigning each noise pixel to the dominant grain in its neighborhood [17]. As shown in Figure 1a, the algorithm assigns the noise pixel (black) to grain A during denoising because it is surrounded by six neighbors from grain A versus only two from grain B. Following the majority principle, the denoising result is shown in Figure 1c. For microstructure with a large average grain size, the substantial disparity between grain size and pixel size means that the assignment of noise pixels has a negligible impact on the measured grain size. However, when the average grain size falls into the tens-to-hundreds of nanometers range, noise becomes pronounced, as illustrated in Figure 1b. Limited resolution can lead to some grains containing only a single pixel. Following the majority principle, noise pixels are very unlikely to be assigned to these small grains during the denoising process, the result is shown in Figure 1d. Humphreys reported that when the indexing rate exceeds 85%, the grain size measurement error due to noise can be limited to below 10% [13]; this condition is often difficult to satisfy for fine-grained microstructures approaching the effective resolution limit.

Denoising also constitutes a fundamental task in computer vision. Established methods include filter-based techniques, wavelet transforms, statistical models, and, more recently, deep learning approaches [18,19,20,21]. DnCNN is recognized as a pioneering CNN-based denoiser [22]. Nevertheless, conventional methods rely on training data generated by artificially corrupting clean images, which limits their generalization to real-world noise. To address this gap, generative adversarial networks (GANs) have been introduced to synthesize training images with noise statistics closer to those of real images, thereby improving performance on practical applications [23,24,25]. Although super-resolution (SR) and denoising have traditionally been treated as separate tasks, recent studies in medical imaging have begun to integrate them, enabling simultaneous SR and denoising of magnetic resonance imaging (MRI) and computed tomography angiography (CTA) data [26,27]. Furat et al. [28] compared the performance of SRResNet, U-NetGAN, SRGAN, and CinCGAN for the SR of lithium-ion scanning electron microscopy (SEM) micrographs. Their results demonstrated that SRGAN yielded the best performance, effectively suppressing image noise and generating the sharpest boundaries. The architecture of SRGAN primarily consists of a generator and a discriminator, incorporating an “adversarial training” mechanism. The generator, based on the SRResNet architecture, aims to reconstruct high-frequency details, while the discriminator is trained to distinguish between real high-resolution images and those synthesized by the generator. Through this adversarial process, the generator is optimized to fit the distribution of natural images, thereby producing super-resolved images with more realistic textures and perceptual quality. Ahmad et al. [29] proposed a deep learning method integrating phase-field simulation, Fourier transform, and attention mechanism, constructed a real-noise microstructure dataset based on optical microscope images, and designed a dedicated denoising network to achieve efficient denoising of material micrographs while preserving critical structures such as grain boundaries.

With the rapid development of deep learning, its application to EBSD analysis has attracted increasing attention in recent years. The application of deep learning in EBSD technology mainly involves two directions. One approach involves employing deep learning for a more precise analysis of Kikuchi bands, which leads to reduced noise and enhanced reliability in EBSD indexing [30,31]. Andrews et al. [31] employed a convolutional denoising autoencoder to preprocess EBSD Kikuchi patterns. This method enhanced Kikuchi band contrast and suppressed image noise, significantly improving the confidence index, angular fitting accuracy, and pattern quality in Hough indexing. Meanwhile, it effectively reduced phantom strain in HR-EBSD measurements, providing a lightweight and compatible solution for high-quality indexing of noisy EBSD data. Winkelmann et al. [32] developed a simulation-driven SR EBSD method. By leveraging high-resolution Kikuchi pattern simulation and supersampling, their approach enables high-precision measurement of relative deformation gradient tensors and strain fields from low-resolution experimental patterns. This technique achieves a significant reduction in data size while maintaining measurement accuracy. The other is to process EBSD data using deep learning, including SR and denoising. Jung et al. [33] employed the SRResNet network to perform SR on low-resolution (LR) microstructure images, utilizing the SR results for microstructure characterization and finite element mechanical analysis. Jangid et al. [34] proposed a quaternion-based orientation recognition and loss function considering rotation effects and crystal symmetries, which reduces orientation errors generated during the SR process. While these methods have advanced the field, they often overlook the noise in EBSD measurements. Such noise often translates into systematic geometric and topological biases, limiting the fidelity of the reconstructed microstructures. Zheng et al. [35] proposed MSA-GAN, a deep learning-based EBSD image inpainting method combining generative adversarial networks and atrous spatial pyramid pooling (ASPP). They used cellular automata to simulate realistic EBSD defects and trained the model to accurately restore missing regions while preserving reasonable grain boundaries and crystallographic orientations. Zheng et al. [36] proposed GTRG, a physics-constrained EBSD inpainting method that integrates adversarial learning and graph neural networks, enabling excellent preservation of crystallographic constraints and grain topology. However, the noise considered in their work mainly refers to large local missing regions caused by imperfect sample preparation. It does not account for the systematic, grain-boundary-distributed noise arising from the limited effective resolution of EBSD measurements. For nanocrystalline or submicron-grained materials, this type of noise can drastically distort the geometry and topological integrity of the microstructure. Despite the above advances, most existing SR and denoising methods for EBSD images rarely fully consider the unique grain boundary noise induced by the effective resolution of EBSD. Although such grain boundary noise is not as noticeable as large-area block defects, its damage to the integrity of the microstructure should not be underestimated. In addition, evaluations in most studies lack a quantitative assessment of microstructural geometry, topology, and grain statistics, and neglect the characterization of fine grains that are equally critical to morphological and topological integrity.

Peak Signal-to-Noise Ratio (PSNR), Structural Similarity Index (SSIM), Feature Similarity Index (FSIM), and Root Mean Square Error (RMSE) are commonly used metrics for assessing the quality of reconstructed images in computer vision [37]. However, in the SR reconstruction of polycrystalline microstructures, these metrics possess intrinsic limitations in fully reflecting reconstruction fidelity. Compared with ground truth microstructures, LR microstructures suffer from both grain shape distortion and topology distortion, especially in small grains. These grains are often visually inconspicuous; for example, the smallest 37% of grains in the microstructure account for only 5% of the total volume [38]. As a result, changes in the geometry and topological structure of such grains exert negligible effects on the overall pixel-level differences, rendering these metrics relatively insensitive to microstructural variations. Nevertheless, the small grains will induce obvious changes in the geometric morphology and topological distribution of microstructures. These characteristics are essential for reliable quantitative EBSD analysis.

In this study, we focus on small grains that are easily overlooked; although they occupy only a small fraction of the microstructure, they significantly influence the overall geometric and topological characteristics. To systematically investigate these effects, effective resolution-induced noise associated with experimental EBSD is incorporated into simulated data, enabling analysis of the impact of resolution and noise on microstructural integrity. Conventional denoising methods used in experimental workflows may introduce bias in geometric and topological descriptors, potentially hindering accurate quantitative characterization. To address these problems, we construct a training framework that combines SRGAN with effective resolution-based noise modeling to achieve joint SR and denoising of EBSD images. This approach aims to improve the geometric and topological fidelity of reconstructed microstructures. To further evaluate its performance, statistical metrics including grain size distribution and grain boundary number distribution are adopted, which shifts the evaluation focus from visual similarity to the recovery of physically meaningful microstructural characteristics critical for quantitative EBSD characterization. Importantly, despite being trained only on simulated data, the proposed SRGAN-based method exhibits favorable performance on experimental EBSD images, indicating its reliable practical applicability and generalization capability.

## 2. Materials and Methods

### 2.1. Experimental Data

The as-received material was a commercially pure nickel rod (Nickel 270, Ni ≥ 99.97%) with a diameter of 10 mm. Cylindrical specimens were first cut to a thickness of ~1.0 mm and then mechanically ground using a sequence of silicon carbide (SiC) abrasive papers (from 240 to 1000 grit) to achieve a final thickness of approximately 0.82 mm. All specimens were ultrasonically cleaned in acetone for 10 min to remove abrasive residues and surface impurities. High-pressure torsion (HPT) was performed under a pressure of 6 GPa for 10 rotations. Following the HPT process, the sample was annealed at 420 °C for 1 h in an argon atmosphere to reach a fully recrystallized state.

For microstructural characterization, the recrystallized sample was mechanically polished using 240 to 1000-grit SiC papers. Subsequently, the samples were prepared for EBSD analysis via electropolishing. The electropolishing was carried out in an electrolyte consisting of 90% methanol and 10% perchloric acid at 30 V and 245 K for 5 s. EBSD observations were conducted at the mid-radius position (0.5 R, where R is the sample radius). An experimental EBSD image was acquired using a scanning electron microscope (SEM, ZEISS Auriga, Oberkochen, Germany) operated at an accelerating voltage of 20 kV, equipped with an EBSD system (C-Swift and Aztec, Oxford Instruments, High Wycombe, UK) using a step size of 100 nm. EBSD data were analyzed using MTEX (Version 5.5) and Channel5 software (version 5.11.20405.0). An iterative noise reduction routine based on the Channel 5 software was employed to clean the EBSD data. The process began with a 6-neighbor orientation correlation algorithm, iterating until the indexing rate plateaued. Subsequently, the neighbor threshold was progressively reduced (e.g., to 5 neighbors, and so on) to refine the data further. This stepwise reduction continued until the noise was completely eliminated.

### 2.2. Simulated Dataset and Training Strategy

A simulated 3D EBSD microstructure was generated using DREAM.3D software (version 6.5.141) [17]. The volume consisted of 512 × 512 × 512 voxels with a resolution of 25 nm/voxel. The distribution parameters were defined with a location parameter (μ) of 0.3 and a shape parameter (σ) of 0.9. From this 3D microstructure, 512 two-dimensional cross-sectional microstructures, each with a resolution of 512 × 512 pixels, were extracted for subsequent analysis. When the distance between the electron beam and the grain boundary is smaller than the effective resolution, the software fails to resolve the correct orientation, resulting in the generation of noise. The pixel size of the ground truth was fixed at 25 nm, and unidentifiable regions at grain boundaries were set to different widths to simulate various effective resolution conditions. For microstructures with an effective resolution of 25 nm, noise was applied to a one-pixel width on both sides of the grain boundary, while for those with a 50 nm resolution, the noise coverage was increased to two pixels on each side. It should be noted that while the simulated EBSD microstructures realistically reproduce experimental resolution and noise, they inevitably lack the full complexity of physical data. First, intragranular orientation gradients are neglected; however, as the analysis focuses on geometric and topological recovery, this omission is acceptable. Second, while noise is modeled based on effective resolution limits, it may not fully capture the spatial non-uniformity and stochastic nature of real noise (e.g., local clustering from instrumental instability or surface contamination).

To investigate the impact of the experimental noise reduction method on the microstructure, an iterative neighborhood filtering strategy, similar to that implemented in Channel 5 software, was applied to the simulated data containing noise. The procedure commenced with an initial cleaning using a 6-neighbor algorithm, iterating until the indexing rate stabilized. Subsequently, the neighborhood range was progressively reduced (e.g., to 5-neighbors) to further optimize the data. This stepwise reduction continued until the noise was completely eliminated.

Noisy LR images are generated by downsampling the noisy HR images via nearest-neighbor interpolation. Nearest-neighbor interpolation serves as a direct approach to downsample images. For each pixel in the LR grid, the corresponding coordinate is mapped back to the HR space, taking the value of the nearest single pixel without interpolation. Given that EBSD pixels denote distinct crystallographic orientations, this method preserves the discrete nature of electron beam scanning and prevents the generation of non-physical, averaged orientations that typically result from linear interpolation techniques. By incorporating the acquisition principles of EBSD and the underlying noise generation mechanisms into the simulated data, the trained model demonstrates good performance on experimental EBSD data.

Each noise-free HR microstructure was segmented into sixteen individual images of size 128 × 128 pixels, yielding a total of 8192 HR images that served as ground-truth labels. The noisy HR images, downsampled to 0.5 and 0.25 of the original resolution, were segmented into 64 × 64 and 32 × 32 images, respectively, serving as LR images. The SRGAN model [39] for 2× SR was trained using paired LR and HR images. The SRGAN training procedure consists of two steps: first, pre-training the generator to maximize pixel-wise accuracy from LR inputs; and second, incorporating a discriminator for adversarial training. This latter stage focuses on refining perceptual quality to generate sharper details.

**Figure 2:**
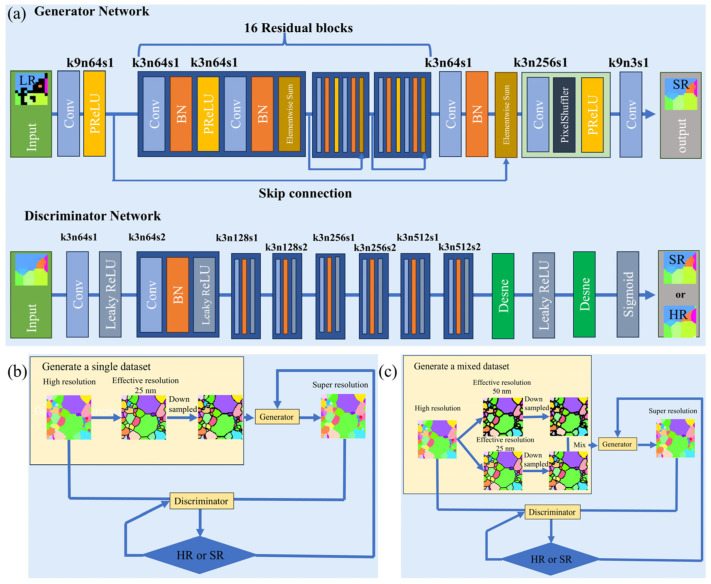
Network architectures and training strategies. (**a**) SRGAN [39] architectures; (**b**) Training strategy based on a microstructure dataset with a single effective resolution of 25 nm; (**c**) Training strategy based on a microstructure dataset with mixed effective resolutions (50% 25 nm + 50% 50 nm). The labels associated with convolutional layers denote the kernel size (*k*), the number of feature maps (*n*), and the stride (*s*). For instance, the label k9n64s1 corresponds to a layer with *k* = 9, *n* = 64, and *s* = 1.

To investigate the impact of the effective resolution of microstructures in the training set on model training, this study constructed two datasets for comparative experiments: one consisting solely of microstructures with an effective resolution of 25 nm, and the other a mixed dataset composed of 50% microstructures with 25 nm resolution and 50% with 50 nm resolution. The SRGAN model shown in Figure 2a was trained using these two strategies; the relevant flowcharts are displayed in Figure 2b,c. In this process, the generator first performs super-resolution reconstruction and denoising on the noisy low-resolution microstructures to yield high-fidelity high-resolution outputs. Subsequently, the discriminator conducts binary discrimination between the generated results and the ground truth. Through this adversarial mechanism, the generator is driven to learn the distribution of finer details, thereby producing realistic images that are difficult to distinguish from the real ones.

For SRGAN training, the generator loss ($L_{G}$) is defined as a weighted combination of three components, namely the image loss ($L_{image}$), adversarial loss ($L_{adversarial}$), and perceptual loss ($L_{perceptual}$)”:

(1) $$L_{G}=\;L_{image}+0.001\times L_{adversarial}+0.006\times L_{perceptual}$$

The image loss is implemented as the mean absolute error (MAE, or L1 loss) between the generated image and the ground truth, which enforces pixel-wise consistency and helps preserve the overall structure, intensity distribution, and low-frequency content. The adversarial loss follows the standard generative adversarial network (GAN) framework and encourages the generator to produce realistic images that are indistinguishable from real samples by the discriminator. It is defined based on the discriminator’s response to generated images, driving the generator to maximize the likelihood of being classified as real. The perceptual loss is computed using high-level feature representations extracted from a pre-trained VGG19 network, which remains fixed during training. By minimizing the L1 distance between feature maps of the generated and ground-truth images, this loss captures semantic structures, edge information, and high-frequency details, thereby improving perceptual quality beyond pixel-level similarity. The weighting coefficients are empirically tuned to achieve a balance between training stability and reconstruction fidelity.

The discriminator is trained to distinguish real images from generated ones, with its loss defined as:

(2) $$L_{D}=L_{real}+L_{fake}$$

where $L_{real}$ and $L_{fake}$ correspond to the discriminator losses on real and generated samples, respectively, following the standard adversarial minimax optimization framework.

The network was implemented via Python 3.6 with the PyTorch 1.10.2 framework. The computer was equipped with an Intel Core i7-13700 CPU, 64 GB RAM (Intel, Santa Clara, CA, USA), and an NVIDIA RTX 3080 GPU (NVIDIA, Santa Clara, CA, USA) for model training and image reconstruction. The specific hyperparameters used to train the network are shown in Table S2.

## 3. Results and Discussion

### 3.1. The Effective Resolution of Experimental and Simulated Data

**Figure 3:**
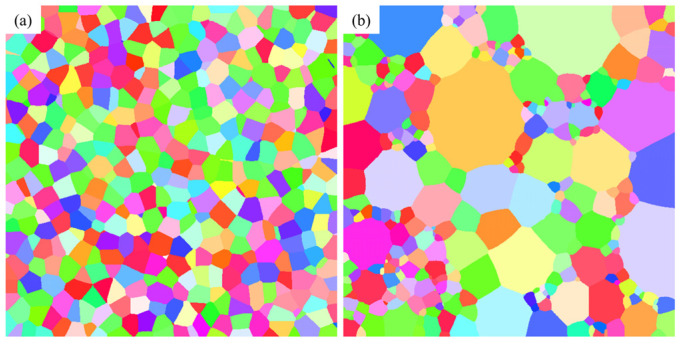
Distribution of boundary in microstructures with the near average grain size (around 780 nm) but different grain size standard deviations. (**a**) Standard deviations = 279.3 nm, (**b**) Standard deviations = 694.4 nm.

Humphreys [13] found that when grain size is significantly larger than effective resolution, the relationship between effective resolution and indexed rate can be expressed as:

(3) $$\Lambda_{A}=\frac{D(1-N_{S})}{4}$$

where $\Lambda_{A}$ denotes the effective resolution in the parallel direction, D is the average grain size, and $N_{S}$ represents the indexed rate. According to the formula, when the effective resolution increases from 25 nm to 50 nm, the indexed rate for data with an average grain size of 780 nm decreases from 87.29% to 74.68%. However, for microstructures with grain sizes in the tens to hundreds of nanometers, the indexed rate is not solely determined by the average grain size but is also strongly influenced by the grain size distribution. As shown in Figure 3, microstructures with an average grain size of approximately 780 nm were generated using the DREAM.3D software, with grain size distribution standard deviations of 279.3 nm and 694.4 nm, respectively. The different degrees of distribution dispersion lead to significant differences in grain boundary density between the two microstructures. Table 1 lists the grain boundary densities and unindexed rates for these two microstructures. Microstructures at various effective resolutions were simulated. At an effective resolution of 25 nm, a two-pixel-wide region along the grain boundaries is covered by noise. At a lower effective resolution of 50 nm, the noise-affected region widens to four pixels. When the grain size distribution is relatively narrow (i.e., smaller standard deviation), the simulation results closely follow the predictions of the Humphreys model (Equation (3)). Since noise induced by limited effective resolution predominantly occurs at the grain boundary, a lower grain boundary density leads to a decreased unindexed rate, thereby resulting in significant deviations from the results calculated using the Humphreys equation.

**Figure 4:**
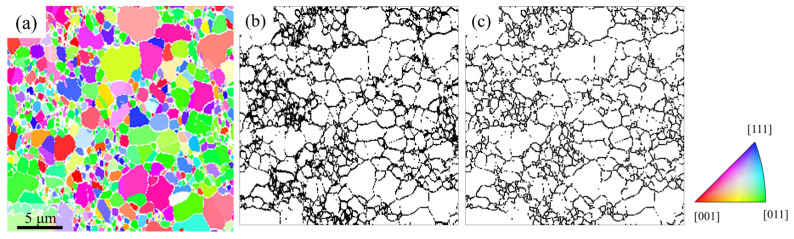
Experimental EBSD image and corresponding noise distribution. (**a**) Raw data. (**b**) Spatial distribution of noise (black) in the EBSD data. (**c**) Noise (black) localized at grain boundary.

The experimental EBSD data shown in Figure 4a exhibits an indexing rate of 82.19%, with an average grain size of 775.1 nm and a standard deviation of 674.4 nm. The unindexed fraction in the data is 17.81%, as illustrated in Figure 4b. In addition to noise distributed along grain boundaries, Figure 4b reveals the presence of block-like noise regions. These block-like features are likely associated with grain loss induced by resolution. Since the simulated data (Figure 3) does not exhibit the blocky noise caused by grain loss, these block-like noise regions were excluded for comparison. The resulting noise distribution image is shown in Figure 4c, where the unindexed rate drops to 12.38%. By comparing the experimental result to the simulated result in Table 1, the effective resolution of the experimental EBSD data is estimated to be approximately 50 nm.

Training SRGAN with simulated data having an average grain size around 780 nm (such as Figure 3) does not accurately capture the block-like noise observed experimentally. To better reproduce this phenomenon, the SRGAN model was trained using simulated microstructures with a smaller average grain size of 282.3 nm, with a pixel size of 25 nm.

**Figure 5:**
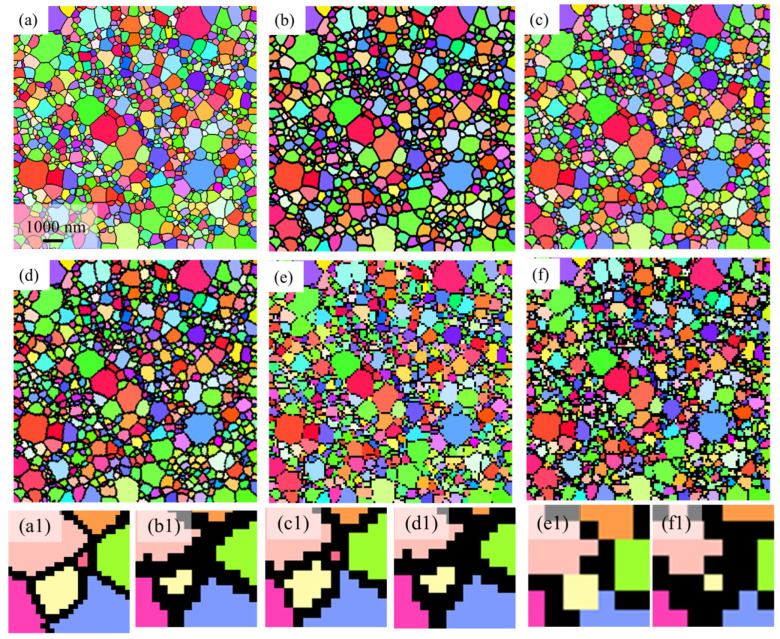
Microstructures at different effective resolutions and downsampling scales. (**a**,**b**) Full-resolution microstructures with effective resolutions of 25 and 50 nm, respectively. (**c**,**d**) Microstructures downsampled to 0.5× at effective resolutions of 25 and 50 nm, respectively. (**e**,**f**) Microstructures downsampled to 0.25× at effective resolutions of 25 and 50 nm, respectively. (**a1**–**f1**) Magnified views of the regions marked in (**a**–**f**), respectively.

For microstructures with an average grain size of 282.3 nm, at an effective resolution of 25 nm, a two-pixel-wide region along the grain boundaries is fully covered by noise, resulting in an indexed rate of 80.75% (Figure 5a). At a lower effective resolution of 50 nm, the noise-affected region expands to four pixels in width, reducing the indexed rate to 60.52% (Figure 5b). The microstructures at 25 nm and 50 nm effective resolutions were then downsampled to 0.5× and 0.25× of the original resolution, as shown in Figure 5c–f. A comparison between the HR and LR microstructures clearly reveals that the reduction in resolution induces serrated grain boundaries. This is mainly due to the insufficient spatial resolution and sparse sampling density of LR imaging. With limited detection units, the system cannot accurately capture the continuous curvature of grain boundaries. Instead, natural smooth boundaries are approximated by segmented outlines, resulting in pronounced stepped and serrated appearances. Thus, such serration is a distortion induced by LR constraints, distinct from the material’s true microstructural characteristics. Figure 5f corresponds to a pixel size of 100 nm and an effective resolution of 50 nm, consistent with the parameters of experiment data. The magnified views of Figure 5a–f are presented in Figure 5(a1–f1), clearly revealing grain deformation and loss induced by the increase in effective resolution and the decrease in image resolution. It can be observed that the block-like noise in Figure 5(f1), similar to that in Figure 4b, is caused by grain loss.

### 3.2. The Effect of Effective Resolution on SR

**Figure 6:**
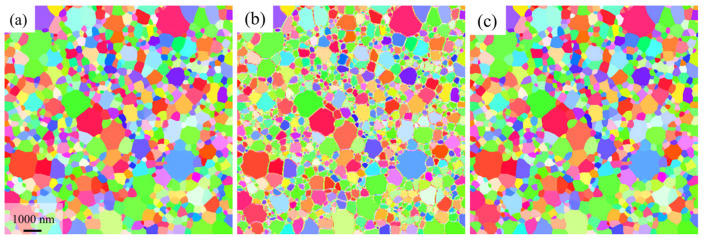
SR (2×) performance with varied effective resolutions and training datasets. (**a**) Effective resolution: 25 nm (trained on 25 nm data). (**b**) Effective resolution: 50 nm (trained on 25 nm data). (**c**) Effective resolution: 50 nm (trained on a mixed dataset of 25 and 50 nm data).

The microstructures with effective resolution of 25 nm and 50 nm were downsampled to 0.25× of the original resolution as test data (Figure 5e,f). Training the network solely on the microstructure with effective resolutions of 25 nm produced the outputs in Figure 6a,b. While the SR model performs well on the 25 nm effective resolution sample (Figure 6a) by achieving simultaneous SR and denoising, applying the same network to 50 nm effective resolution microstructures introduces significant artifacts, manifesting as randomly colored pixels along grain boundaries (Figure 6b). This indicates that a mismatch between the effective resolution of the test data and the training set leads to performance degradation. When the model is trained only at a single effective resolution (25 nm), it learns a fixed width for the unindexed noise regions at grain boundaries under that specific resolution. However, when the model is directly applied to 50 nm data, the actual unindexed noise region at grain boundaries becomes wider. The mismatch between the noise scale learned during training and the actual noise width at 50 nm effective resolution breaks physical correspondence. Consequently, the model fails to adapt to the expanded noise pixel range at grain boundaries, leading to obvious artifacts. Training on a mixed dataset containing both 25 nm and 50 nm microstructures substantially improves the model’s generalization, as shown in Figure 6c. This indicates that the effective resolution of the training data critically influences SR performance.

### 3.3. SRGAN Performance on the Simulated Image

**Figure 7:**
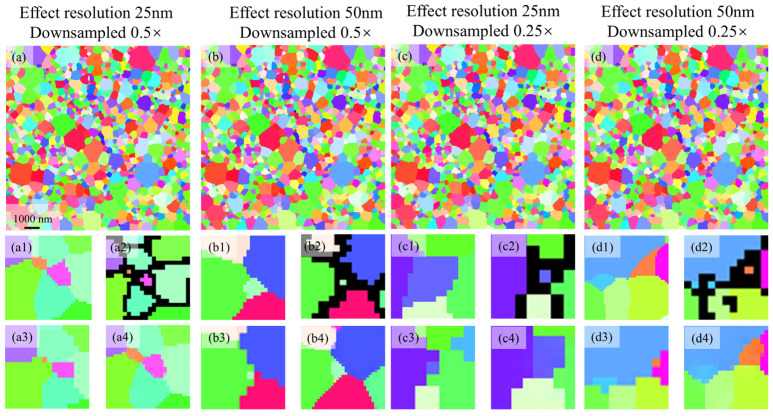
SR results for microstructures downsampled by factors of 0.5× and 0.25×. (**a**–**d**) 2× SR outputs for microstructures with effective resolutions of 25 nm and 50 nm. (**a1**–**d4**) Localized comparisons: (**a1**,**b1**,**c1**,**d1**) Ground truth, (**a2**,**b2**,**c2**,**d2**) noisy LR, (**a3**,**b3**,**c3**,**d3**) experimental denoiseing result and (**a4**,**b4**,**c4**,**d4**) SR reconstructed.

For microstructures downsampled to 0.5× and 0.25× of the original resolution, SRGAN was employed to perform 2× SR. Quantitative evaluation of SRGAN-reconstructed microstructures across downsampling factors and effective resolutions using standard computer vision metrics (as shown in Table S1). Figure 7 presents the SR results for the low-resolution microstructure shown in Figure 5c–f, demonstrating that the model effectively achieves denoising during the SR process. By comparing the locally magnified details of the experimental denoising method (Figure 7(a3,b3,c3,d3)) with those of the SRGAN (Figure 7(a4,b4,c4,d4)), it is evident that the SRGAN more effectively recovers the morphology and size of the single-pixel grains (Figure 7(a2,b2,c2,d2)) relative to the ground truth (Figure 7(a1,b1,c1,d1)). Furthermore, it is observed that the experimental denoising method alters the grain topology. In Figure 7(a3,b3,c3,d3), the topology structure of single-pixel grains, which are clearly presented in the ground truth images Figure 7(a1, b1, c1, d1), fails to be accurately restored. The single-pixel grain in Figure 7a3 exhibits only 2 grain boundaries, whereas the ground truth in Figure 7a1 clearly displays 5. Similarly, the single-pixel grains in Figure 7(b3, c3, d3), present merely 2 boundaries, while the corresponding ground truth images Figure 7(b1, c1, d1).distinctly show 3 boundaries each. Moreover, changes in the topological structure of one grain can propagate to adjacent grains, consequently impacting the global topological connectivity of the microstructure. In contrast, the SRGAN successfully restores the original topology of these grains. 

The grain statistics under different downsampling ratios and effective resolutions are summarized in Table 2. Using the HR microstructure (1152 grains) as a reference, a consistent reduction in detected grain number is observed in all LR conditions. At a downsampling ratio of 0.5×, the grain retention rate decreases from 89.5% at 25 nm effective resolution to 80.8% at 50 nm, corresponding to a grain loss of 10.5% and 19.2%, respectively. When more aggressive downsampling (0.25×) is applied, the retention further decreases to 81.9% and 71.9%, indicating a pronounced accumulation of structural information loss. Across all cases, degradation in effective resolution introduces an additional ~9–10% grain loss, while downsampling contributes a further ~7–9% reduction. This systematic loss of small grains leads to a biased grain size distribution, with a preferential suppression of fine-grained features that are critical to microstructural statistics. The Hall–Petch relationship dictates that finer grains enhance strength by impeding dislocation motion. However, the systematic loss of tiny grains artificially inflates the average grain size. This leads to an underestimation of grain boundary strengthening and inaccurate mechanical predictions.

**Figure 8:**
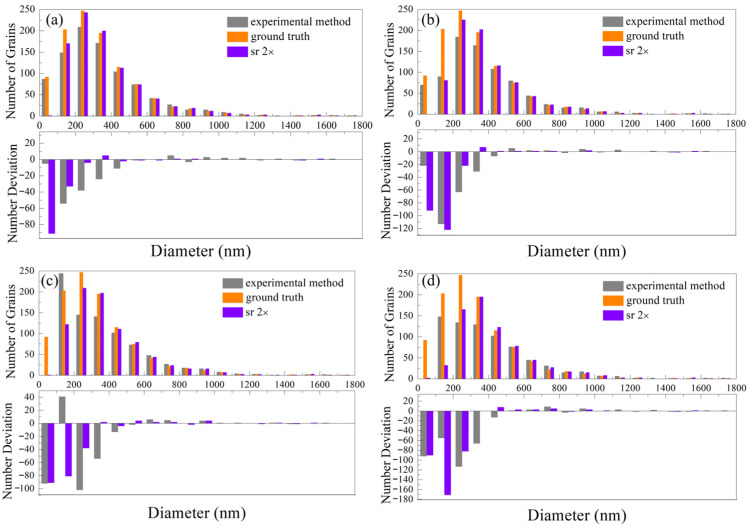
Comparison of grain size distributions from LR data processed by different methods. Downsampling scale 0.5×: (**a**) effective resolution = 25 nm; (**b**) effective resolution = 50 nm. Downsampling scale 0.25×: (**c**) effective resolution = 25 nm; (**d**) effective resolution = 50 nm. Subfigure consists of two vertically arranged panels: the upper panel shows the grain size distribution, while the lower panel quantifies the deviation from the ground truth across all bins. Grain size statistics were obtained from a single large-area map with sufficient grain numbers.

As shown in Figure 8, the grain size distributions of the ground truth and two denoising methods are compared. Grain size statistics were derived from a single large-area map, which contains a sufficient number of grains to ensure statistical representativeness. Each subfigure consists of two vertically arranged panels: the upper panel shows the grain size distribution histogram, while the lower panel quantifies the deviation of each grain size bin from the ground truth. A comparative analysis is conducted on microstructures with effective resolutions of 25 nm and 50 nm under two downsampling scales (0.5× in Figure 8a,b, and 0.25× in Figure 8c,d).

Theoretically, grain loss induced by resolution degradation and noise interference is expected to first affect the smallest grains. However, compared with the ground truth, the experimental denoising results show a significant reduction in medium- and small-sized grains, even before the complete disappearance of the finest grains. As shown in Table 2, for the case of 25 nm effective resolution with 0.5× downsampling, a total of 121 grains are lost. According to Figure 8a, only 5 grains are removed in the smallest size bin (0–100 nm), whereas 54, 38, 24, and 11 grains are lost in the second to fifth bins, respectively. This abnormal phenomenon becomes more severe as the resolution decreases and the effective resolution increases.

Figure 8b shows the grain size distribution of different denoising methods at 0.5× downsampling with an effective resolution of 50 nm. As indicated in Table 2, this condition results in a total loss of 221 grains. The ground truth contains 92 grains in the smallest size bin, which should be largely eliminated. Nevertheless, the experimental denoising method only reduces 22 grains in this bin, whereas the cumulative loss from the second to the fifth smallest bins reached 214.

This phenomenon originates from the noise allocation mechanism of the experimental method described above. Noisy pixels tend to be preferentially assigned to adjacent grains with larger surrounding pixel counts. Consequently, numerous fine grains whose sizes are underestimated due to noise, particularly the single-pixel grains shown in Figure 7, cannot recover their true dimensions during denoising and instead accumulate abnormally in the smallest size bin. As a result, partial grains originally located in the third and fourth size bins of the ground truth are misclassified into the first and second smallest bin due to noise disturbance.

When the downsampling scale decreases to 0.25×, the pixel size increases to 100 nm. The equivalent circular diameter of the smallest identifiable single-pixel grain in the microstructure is approximately 112 nm, exceeding the smallest grain size bin (0–100 nm). These single-pixel grains, whose original sizes cannot be restored during denoising, accumulate predominantly in the second smallest grain size bin. Accordingly, this bin replaces the third-smallest bin and becomes the peak region of grain count. As a result, the grain size distribution obtained by experimental denoising is severely distorted, deviating markedly from the log-normal distribution of the ground truth and exhibiting an exponential-like tendency, as shown in Figure 8c,d.

In contrast, the SRGAN model performs SR reconstruction and denoising simultaneously. Specifically, each noisy pixel in the low-resolution image is upsampled into four pixels after 2× SR. During training, SRGAN learns the intrinsic mapping between noisy microstructures and their corresponding ground truth. Consequently, noisy pixels can be more reasonably and evenly distributed among adjacent grain regions (Figure 7), preventing over-processing and artificial structural damage to small grains. This mechanism effectively mitigates microstructural distortion induced by noise reduction and resolution reconstruction. As a result, grain contours, morphological details, and intrinsic grain size distribution characteristics are well preserved. Overall, SRGAN-based reconstruction captures the physically expected grain-loss behavior, in which the smallest grains are preferentially lost, resulting in grain size distributions that are closest to the ground truth.

Since grain loss is primarily concentrated within the two smallest size bins (0–200 nm), the number of grains larger than 200 nm remains relatively stable. Therefore, the extent of grain size shift is evaluated by analyzing the number of grains larger than 200 nm under different denoising methods. Specifically, the relative deviation is defined as the difference between the number of grains larger than 200 nm in the denoised results and that in the ground truth, normalized by the corresponding ground-truth value. As shown in Table 3, the relative deviation of grains larger than 200 nm is significantly higher for the conventional experimental denoising method compared with SRGAN across all conditions. For the experimental method, the deviation increases from 8.72% to 23.2% as the downsampling scale decreases and the effective resolution deteriorates, indicating a pronounced shift in grains toward smaller size bins. In contrast, SRGAN consistently exhibits much lower deviations (0.1–8.1%), demonstrating a substantially improved preservation of grain size distribution. Although both methods show increasing deviation with decreasing resolution, the growth rate is significantly lower for SRGAN, suggesting enhanced robustness against resolution degradation. Across four data, experimental denoising methods cause an average of approximately 16.03% of the grains to shift toward smaller size bins, whereas the SRGAN approach reduces this proportion to about 3.35%. These results confirm that the proposed method effectively mitigates grain size redistribution and preserves the statistical integrity of microstructures.

**Figure 9:**
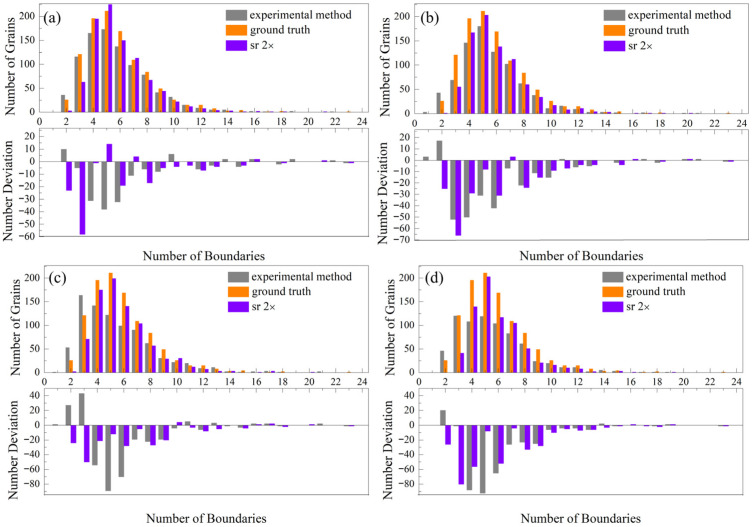
Grain boundary number distributions for LR data processed by different methods at reduced resolutions. Downsampling scale 0.5×: (**a**) effective resolution = 25 nm; (**b**) effective resolution = 50 nm. Downsampling scale 0.25×: (**c**) effective resolution = 25 nm; (**d**) effective resolution = 50 nm. Subfigure consists of two vertically arranged panels: the upper panel shows the grain boundary number distribution, while the lower panel quantifies the deviation from the ground truth across all bins.

Noise and resolution degradation not only alter grain size but also change the topological connectivity between grains, thereby affecting the grain boundary number. Figure 9 summarizes the effects of different denoising methods on the grain boundary number distribution. Each subfigure consists of two vertically arranged panels: the upper panel presents the grain boundary number distribution, while the lower panel quantifies the numerical deviation of each bin from the ground truth.

Figure 9a,b show the results with effective resolutions of 25 nm and 50 nm, respectively. As listed in Table 2, after downsampling to 0.5× resolution, a total of 121 grains are lost in the 25 nm effective resolution case, and 221 grains disappear in the 50 nm case.

According to Lewis’s law [40], an empirical linear relationship exists between grain size and the number of grain boundaries. The quantitative expression of Lewis’s law is:

(4) $$\bar{A}\left(n\right)=a\times n+b$$

where $\bar{A}\left(n\right)$ is the average area of n-sided grains, n is the grain boundary coordination number, and a, b are structural constants. This indicates that smaller grains typically possess fewer grain boundaries. Such grains are more susceptible to disappearance under noise and resolution degradation. However, as shown in Figure 9a, the number of grains with 2–3 boundaries does not decrease significantly after experimental denoising. In contrast, grains with 4, 5, and 6 boundaries decrease markedly. This suggests that grains originally possessing 4–6 boundaries are likely transformed into grains with fewer boundaries (2–3) due to boundary loss induced by noise and resolution degradation, which is consistent with the observations in Figure 7.

The experimental denoising method results in a flatter distribution of grain boundary counts, characterized by a leftward shift in the peak’s horizontal coordinate and a decrease in its vertical coordinate. This phenomenon is particularly evident in Figure 9c,d. By contrast, the denoise based on SRGAN effectively recovers the grain boundaries of fine grains, as reflected in Figure 7. It greatly alleviates the abnormal transformation of 4–6 sided grains into 2–3 sided small grains induced by noise and resolution degradation. Accordingly, the original distribution characteristics of grain boundary number are well preserved to a certain degree.

To quantitatively compare the topological characteristics of grain boundary number distributions, the peak coordinate of each distribution, and the grain boundary percentage are extracted and summarized in Table 4. In the ground truth microstructure, the distribution consistently peaks at 5-sided grains with a frequency of 211, indicating that 5 is the dominant grain boundary coordination number.

For the experimental denoising method, the peak position remains unchanged at 5 under the 0.5× downsampling scale, although the peak intensity decreases significantly. However, when the resolution is reduced to 0.25×, the peak position shifts from 5-sided grains to 3-sided grains, accompanied by a sharp drop in peak intensity.

In contrast, the SRGAN-based method better preserves grain boundary information of small grains, resulting in total boundary counts and distributions closer to the ground truth. On average, SRGAN recovers 2.58% more grain boundaries than the experimental method. Moreover, the peak position remains consistently at 5-sided grains across all downsampling scales, matching the dominant coordination number of the original microstructure. These results demonstrate that SRGAN more effectively preserves both the quantity and statistical distribution of grain boundary coordination numbers, thereby maintaining the topological integrity of the microstructure.

### 3.4. SRGAN Performance on the Experimental Image

**Figure 10:**
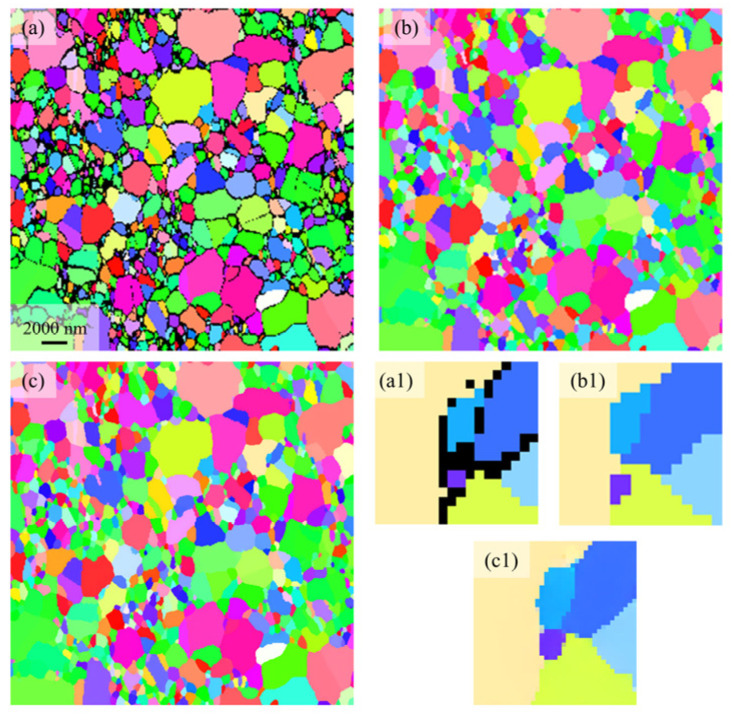
SR reconstruction of experimental EBSD image. (**a**) original low-resolution image, (**b**) experimental denoise, (**c**) 2× SR. (**a1**–**c1**) Magnified views of the regions indicated in (**a**–**c**).

Using a network trained on a mixed dataset comprising microstructures with effective resolutions of 25 nm and 50 nm, 2× SR was applied to the experimental EBSD image, as shown in Figure 10. Although the model was trained on simulated images, it generalizes well to the experimental image. As shown in Figure 10(a1), the small grain (purple) should have 3 or 4 adjacent grains. However, after denoising using Channel 5 software, this grain retains only 2 neighbors. In contrast, the SRGAN method restores the grain boundary count to 4, which is consistent with the observations in Figure 7.

**Figure 11:**
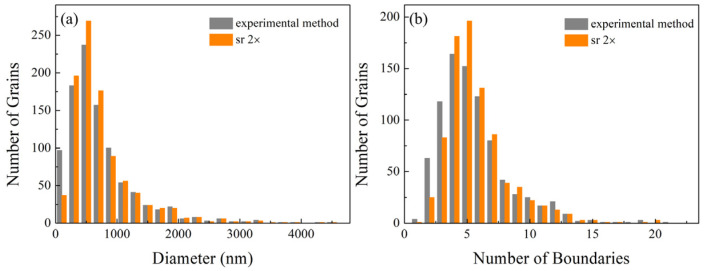
Comparison of grain size and grain boundary distributions from the experimental EBSD image processed using different methods. (**a**) Grain size distributions, (**b**) boundary distributions.

A comparative analysis of grain size distributions across different denoising methods is presented in Figure 11a, while the corresponding grain boundary distributions are shown in Figure 11b. The SRGAN-based method effectively suppresses the shift in grain sizes and grain boundary counts toward smaller bins, thereby reducing statistical distortion. In the experimental denoising method, small grains cannot obtain their own noise pixels, which makes their measured size smaller than the actual size. This results in the redistribution of grains originally located in the second to fourth bins toward smaller bins. In contrast, SRGAN enables a more balanced allocation of pixel information, thereby alleviating this effect.

At the same time, due to the inability of small grains to obtain their corresponding noise pixels, their topological structure will also change. As shown in Figure 10, grains originally possessing three or four grain boundaries are reduced to only two grain boundaries after denoising by the experimental method, leading to a shift in the grain boundary count distribution toward smaller bins and a reduction in the peak from 5 to 4 (Figure 11b). In contrast, denoising with SRGAN can effectively alleviate this problem. Although the true microstructure cannot be directly obtained due to the limitations of experimental characterization techniques, the SRGAN-denoised results show good consistency with the trends reported in Figure 8 and Figure 9.

Despite the promising performance of the proposed method, several limitations should be acknowledged. First, the true ground truth microstructure of experimental EBSD data cannot be directly accessed, which limits fully quantitative validation against real microstructures. This limitation fundamentally arises from a scale mismatch in available characterization techniques. The experimental datasets used in this study exhibit a grain size distribution ranging from approximately 100 nm to 4.7 μm, which lies near the practical resolution limit of EBSD. While higher-resolution techniques such as Transmission Kikuchi Diffraction (TKD) and Transmission Electron Microscopy (TEM) can resolve finer structural details, their limited field of view makes it extremely challenging to acquire continuous microstructural maps over sufficiently large areas for such grain sizes. Therefore, further evaluation on a wider range of experimental EBSD datasets with varying grain sizes and microstructural characteristics is required to better assess the generalizability of the method.

Second, the current model is trained using microstructures with effective resolutions of 25 nm and 50 nm. Although no assumptions specific to a particular material system are introduced during dataset construction, the framework is primarily constrained by the effective resolution and noise characteristics inherent to EBSD measurements. However, the present experimental validation is limited to nickel-based EBSD data, which may not fully capture variability across different material systems, thereby potentially limiting the statistical completeness of the evaluation and the assessment of inter-material differences.

Extending the method to other materials or broader effective resolution regimes would require enriching the training dataset with additional representative microstructures, followed by retraining and further validation.

## 4. Conclusions

This study presents a machine learning framework based on SRGAN, which integrates an effective resolution-based noise model to capture key characteristics of EBSD measurements. The proposed framework facilitates simultaneous super-resolution reconstruction and denoising under limited-resolution and noisy conditions, improving geometric and topological consistency in microstructural representations.

Quantitative analysis shows that experimental denoising methods cause an average of approximately 16.03% of the grains to shift toward smaller size bins, whereas the SRGAN-based approach reduces this proportion to about 3.35%. In addition, it reconstructs 2.58% more grain boundaries on average and reduces distribution distortions introduced by conventional approaches. These results demonstrate the superior statistical consistency and structural fidelity of the proposed framework.

## Figures and Tables

**Table 1:** Grain boundary density and unindexed rates for two microstructures with similar average grain sizes around 780 nm, but different standard deviations at various effective resolutions.

Effective Resolution (nm)	Average Grain Size (nm)	Standard Deviations (nm)	Grain Boundary Density (nm^−1^)	Simulated Unindexed Rate (%)	Calculated Unindexed Rate (%)
25	783.7	279.3	2157.9	10.79	12.77
50	783.7	279.3	2157.9	20.83	25.57
25	785.5	694.4	1270.6	6.35	12.73
50	785.5	694.4	1270.6	12.25	25.56

**Table 2:** Grain statistics deviation under different downsampling ratios and effective resolutions.

Down Resolution	Effective Resolution	HR	LR	Grain Retention Rate (%)	Grain Loss Rate (%)
0.5×	25 nm	1152	1031	89.5	10.5
	50 nm	1152	931	80.8	19.2
0.25×	25 nm	1152	944	81.9	18.1
	50 nm	1152	828	71.9	28.1

**Table 3:** Relative deviation of grain counts (>200 nm) under different denoising methods.

Down Resolution	Effective Resolution	Experimental Method	SRGAN
0.5×	25 nm	8.72%	0.1%
	50 nm	11.7%	1.2%
0.25×	25 nm	20.5%	4%
	50 nm	23.2%	8.1%

**Table 4:** Peak coordinate and grain boundary percentage (P_GB_), under various conditions.

Down Resolution	Effective Resolution	Processing Condition	Peak Coordinate	P_GB_
0.5×	25 nm	Ground truth	(5, 211)	1
		Experimental	(5, 173)	87.6%
		SRGAN	(5, 225)	89.0%
0.5×	50 nm	Ground truth	(5, 211)	1
		Experimental	(5, 180)	77.5%
		SRGAN	(5, 203)	79.6%
0.25×	25 nm	Ground truth	(5, 211)	1
		Experimental	(3, 164)	77.0%
		SRGAN	(5, 199)	81.0%
0.5×	50 nm	Ground truth	(5, 211)	1
		Experimental	(3, 120)	66.9%
		SRGAN	(5, 203)	69.7%

## Data Availability

Data will be made available on request.

## Supplementary Materials

The following supporting information can be downloaded at: https://www.mdpi.com/article/10.3390/nano16100583/s1, Table S1: Quantitative evaluation of SRGAN-reconstructed microstructures across downsampling factors and different effective resolutions. Table S2: Hyperparameters used for our network.
