# Supplementary material for: SRGAN-Based Joint Super-Resolution and Denoising for Mitigating Geometric and Topological Biases in Fine-Grained Electron Backscatter Diffraction Images
Source: Nanomaterials (Basel). 2026 May 10;16(10):583. doi: 10.3390/nano16100583 (PMC13209600; doi:10.3390/nano16100583)
Supplement: Supplementary file 1 [file nanomaterials-16-00583-s001.zip › nanomaterials-4293350-supplementary.pdf]

Table S1. Quantitative evaluation of SRGAN-reconstructed microstructures across downsampling factors and different effective resolutions.

downsampling factors	effective resolution	psnr	ssim	fsim	rmse
0.5×	25nm	24.059	0.9369	0.9106	15.9805
0.5×,	50nm	20.928	0.8807	0.8534	22.916
0.25×	25nm	17.712	0.7545	0.6918	33.1849
0.25×	50nm	16.372	0.6977	0.6403	38.7194

Pseudocode of SRGAN

Algorithm: Integrated EBSD Super-Resolution and Denoising Based on SRGAN
Input: Low-resolution EBSD images (LR) with resolution-induced noise
High-resolution EBSD images (HR) as ground truth
Output: High-quality super-resolved EBSD images (SR)

Initialize:
Generator G (for super-resolution and denoising)
Discriminator D (for adversarial training)
Loss function: Content loss + Adversarial loss + MAE loss
Optimizer: Adam for both G and D

Training Process:
For each epoch in total training epochs:
For each batch of training data:
1. Input LR EBSD images into Generator G
2. Generator outputs super-resolved images SR
3. Compute loss between SR and HR:
   - Content loss (feature-level similarity)
   - Adversarial loss from Discriminator D
   - Pixel loss (MAE) for noise suppression
4. Update Generator G to minimize total loss
5. Update Discriminator D to distinguish real HR and fake SR
6. Save model with optimal performance

Inference Process:
Load trained Generator G
For each test low-resolution EBSD image:
Generate high-resolution, denoised output SR

Table S2. Hyperparameters used for our network

Hyperparameter	Value
Upscale factor	2
Training epochs	100

Batch size	16
Optimizer	Adam
Learning rate	0.001
Data workers	8
Loss function	adversarial + image + perception
Evaluation metrics	PSNR, SSIM, MAE

Github link for code and demo: https://github.com/SpursLD/code-and-demo-for-nano-materials
